# Embracing Digital Transformation: A Three-Cycle Audit on Improving Intraoperative Tourniquet Documentation Following BOAST (British Orthopaedic Association Standards for Trauma) Guidelines

**DOI:** 10.7759/cureus.65652

**Published:** 2024-07-29

**Authors:** Abdul-Hadi Kafagi, Marwan Tahoun, Anand Pillai

**Affiliations:** 1 Trauma and Orthopaedics, Wythenshawe Hospital, Manchester University NHS Foundation Trust, Manchester, GBR; 2 Trauma and Orthopaedics, University of Manchester Medical School, Manchester, GBR

**Keywords:** orthopaedic surgery, electronic patient records, operative records, intraoperative tourniquet, boast

## Abstract

Introduction

Tourniquets are vital devices in orthopaedic surgery that aid in creating a bloodless field. While they reduce operative time and outcomes, improper use can lead to severe complications. The British Orthopaedic Association Standards for Trauma (BOAST) offers guidelines for safe intraoperative tourniquet usage, emphasising proper application and accurate documentation. With the push for a paperless National Health Service (NHS), orthopaedic units across the UK are transitioning to electronic patient records (EPR). In this audit, we aim to evaluate the impact of electronic patient records (EPR) implementation on tourniquet documentation and subsequent interventions to enhance guideline adherence.

Methods

This closed-loop audit evaluated 159 emergency trauma cases at a single UK-based district general hospital across three different cycles. The first cycle (n=50) was collected before the trust-wide adoption of an EPR system (EPIC HIVE). Upon collecting the second cycle (n=59), local intraoperative tourniquet usage results compared to BOAST were presented at a departmental audit meeting. A specialised smart phrase template was subsequently shared with the department and the results were re-audited in the third cycle (n=50). Statistical analyses were performed to compare the cycles.

Results

Following the introduction of the EPR, compliance with documentation standards (fully and partially adhered to) initially declined from 38.0% to 17.0%. Post-intervention, compliance increased to 62.0% (p=0.0005). Individual aspects of documentation revealed notable improvements, including increases in skin assessment pre- and post-tourniquet application (0% to 56% and 0% to 60%, respectively, p<0.0001), isolation method (0% to 60%, p<0.0001), exsanguination method (2% to 24%, p=0.0003), tourniquet pressure (39% to 76%, p=0.0001) and tourniquet time (59% to 94%, p=0.0001). Lower limb tourniquet pressures significantly decreased between audit cycles (mean: 287.06 vs mean: 265.91, p=0.007) while upper limb pressures remained relatively stable (mean: 236.67 vs mean: 236.56, p=0.993). No tourniquet-related complications were reported across all audit cycles.

Conclusion

While the introduction of new EPR systems may initially present challenges in upholding accurate operative records, the incorporation of specialised electronic templates and active staff engagement has shown to be instrumental in improving tourniquet safety and enhancing care standards.

## Introduction

Pneumatic tourniquets are ubiquitous in extremity surgery, providing surgeons with an optimal intraoperative environment by creating a bloodless field. Primarily functioning as external devices, tourniquets compress and occlude underlying vascular structures to reduce blood flow. The pressure exerted by the tourniquet must exceed arterial blood pressure to achieve successful cessation of blood flow. Additionally, this containment of blood flow aids in confining anaesthetics within the extremity, preventing their spread centrally, which is particularly beneficial during regional anaesthesia and nerve blocks. Notably, the use of tourniquets has been associated with significant reductions in operative time and subsequent blood loss [1].

Despite their advantages, tourniquets carry rare yet grave complications, including thrombus formation, nerve damage, compartment syndrome, pressure sore development and burn injuries [2]. These complications are invariably linked to improper application, prolonged use [3] and excessively high pressure settings [4].

To mitigate such risks, the British Orthopaedic Association Standards for Trauma (BOAST) released comprehensive guidelines to ensure the safe use of intraoperative tourniquets [5]. These guidelines highlight situations warranting tourniquet usage and prescribe recommended tourniquet width, pressure levels, and duration of use during surgery. Additionally, the guidelines emphasise the necessity of accurate documentation in the operative records, outlining four essential components and advising adherence to patient-specific pressure thresholds based on systolic blood pressure. These components include pre- and post-tourniquet application skin assessments, methods of isolation and exsanguination, and precise documentation of tourniquet pressures and ischaemic time. Moreover, the guidelines provide protocols for managing extended tourniquet times and suspected complications such as burns, nerve damage, or ischaemia resulting from tourniquet use.

The modernisation of patient records marks a profound paradigm shift, characterised by the transition from traditional paper-based systems to electronic patient records (EPR). The adoption of electronic systems represents a new frontier in healthcare documentation, offering the promise of streamlined processes and improved access to patient data. However, the initial hurdles of staff training and system integration may inadvertently affect compliance with emerging documentation protocols. As the healthcare sector works towards a paperless National Health Service (NHS) [6], orthopaedic units nationwide face challenges in maintaining documentation standards amidst this transformation.

This audit seeks to evaluate the impact of EPR implementation on documentation practices of intra-operative tourniquet use within a major orthopaedic unit and aims to assess the effectiveness of subsequent interventions designed to enhance adherence to BOAST guidelines.

## Materials and methods

This retrospective clinical audit focused on emergency cases treated at a regional orthopaedic unit within a district general hospital registered with the local audit under code S290.

The audit adhered to BOAST guidelines governing the safe application of intraoperative tourniquets. This guidance highlights essential parameters that should be considered and documented in the operative records, as outlined in Table 1.

**Table 1:** Summary of audit standard parameters: modified BOAST guidelines for safe intraoperative tourniquet use BOAST: British Orthopaedic Association Standards for Trauma

Audit Standard Parameters
Documentation of quality of skin pre- and post-tourniquet application
Documentation of the method of isolation used to exclude skin preparation fluids from seeping under tourniquet
Documentation of method to achieve successful exsanguination
Documentation of the duration of tourniquet use
Ischaemic time should be less than 120 minutes
Documentation of tourniquet pressure
<16 years: Limb occlusion pressure plus 50 mmHg OR systolic blood pressure plus 50-100 mmHg
>16 years: Systolic blood pressure plus 70-130 mmHg for the lower limb and 50-100 mmHg for the upper limb

The audit comprised three cycles: an initial cycle spanned from December 2021 to March 2022, utilising paper operative notes before the trust-wide adoption of the EPR (EPIC HIVE) in September 2022. Subsequent cycles were conducted from June 2023 to July 2023 (second cycle) and October 2023 to November 2023 (third cycle). Patient data were collected from consecutive upper and lower limb surgeries where tourniquet use was anticipated. Recorded information included patient age, operative procedure, explicit documentation of tourniquet usage, pre- and post-tourniquet skin condition, isolation and exsanguination methods as well as tourniquet pressure and duration. In cases where tourniquet pressures were mentioned in the operative record, adherence to BOAST guidelines was categorised into three groups: 'Fully adhered' indicated that tourniquet pressure and all recommended aspects of tourniquet use were recorded as per guidelines. 'Partially adhered' meant tourniquet pressure was in line with guidelines, and three or more aspects of BOAST guidance were recorded. 'Not adhered' indicated deviation from the guidelines, with tourniquet pressure not meeting criteria and two or fewer aspects of use documented in the operative record. Each aspect of the guidelines was evaluated for its significance based on clinical impact, with tourniquet pressure being prioritised due to its critical role in patient safety and surgical outcomes. Other aspects were considered equally significant for ensuring comprehensive guideline adherence. Data were recorded in Microsoft Excel version 16 (Microsoft Corporation, Redmond, WA, US), and statistical analysis was performed with SPSS for Mac version 29 (IBM Corp., Armonk, NY, US) using the independent samples t-test and chi-square test.

Before the second cycle, a survey was disseminated to 50 registrars and consultants in the orthopaedics department to assess awareness of the BOAST guidelines. Department-wide email communication and informative posters in operating rooms were used to raise awareness and promote adherence. Before the third cycle, audit findings were presented at the Trauma and Orthopaedics Audit for Compliance and Education Meeting. A tailored smart phrase for operative note templates was created, including drop-down options covering all aspects recommended by BOAST. Additionally, the smart phrase would automatically calculate the patient's mean, minimum and maximum systolic blood pressure in the past 24 hours, reminding surgeons to adhere to BOAST guidance for tourniquet pressure calculation before the procedure.

## Results

**Figure 1:**
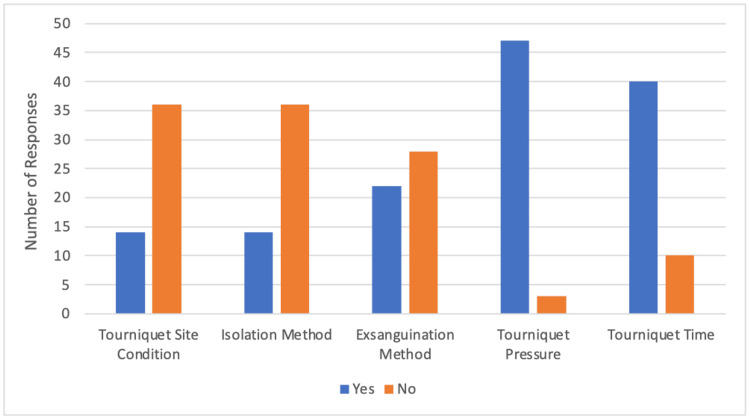
Orthopaedics department survey results: awareness and documentation of BOAST intraoperative tourniquet guidelines BOAST: British Orthopaedic Association Standards for Trauma

Out of the 50 registrars and consultants surveyed in the orthopaedics department, 44% (n=22) were aware of the BOAST intraoperative tourniquet use guidelines. As outlined in Figure 1, the survey revealed limited awareness and documentation practices for certain aspects of the guidelines. Only 28% (n=14) of operating surgeons were aware of documenting the tourniquet site condition before and after the procedure, as well as the isolation technique used to keep the tourniquet dry. Awareness of documenting the exsanguination method was reported by 44% (n=22) of professionals. In contrast, a higher percentage of professionals were aware of the need to document tourniquet pressure and duration, with 94% (n=47) and 80% (n=40), respectively.

In the first audit of 50 patients, only 6.0% (n=3) fully adhered to tourniquet guidelines while 32.0% (n=16) partially adhered, and 62.0% (n=31) didn't comply. By the second cycle with 59 patients, full adherence dropped to 0% (n=0), partial to 17.0% (n=10), and non-adherence increased to 83.0% (n=49). However, in the third cycle with 50 patients, full adherence rose to 18.0% (n=9), partial to 44.0% (n=22), and non-adherence decreased to 38.0% (n=19). Overall, combining full and partial adherence, compliance rates were 38.0%, 17.0%, and 62.0% across the three cycles, respectively. Despite an initial dip, adherence notably improved in the third cycle as seen in Table 2.

**Table 2:** Overall compliance with BOAST recommendations BOAST: British Orthopaedic Association Standards for Trauma

	Cycle 1	Cycle 2	Cycle 3
Number of Patients (n)	50	59	50
Fully Adhered, n (%)	3 (6.0)	0 (0.0)	9 (18.0)
Partially Adhered, n (%)	16 (32.0)	10 (17.0)	22 (44.0)
Not Adhered, n (%)	31 (62.0)	49 (83.0)	19 (38.0)

The baseline characteristics of the patients are outlined in Table 3. They highlight similarities in age and types of operative cases across the audit cycles.

**Table 3:** Baseline characteristics and operation types

	Cycle 2	Cycle 3
Number of Patients (n)	59	50
Mean Age (SD)	48.8 (18.9)	50.3 (21.1)
Age Range	6-88	5-82
Upper Limb: Lower Limb	17:42	18:32
Open Reduction Internal Fixation	37	36
Primary Joint Replacement	6	5
Ligament or Tendon Repair	7	3
Wound Debridement	1	2
Amputation	3	1
Removal of Metalwork	3	2
Fusion Surgery	2	1

Overall, there were significant improvements in skin condition documentation (p=0.0001, p<0.0001), isolation (p<0.0001) and exsanguination methods (p=0.0003), as well as tourniquet pressure (p=0.0001) and time documentation (p=0.0001) in the re-audit, as highlighted in Table 4.

**Table 4:** Rates of documented audit parameters (*) significant p-value (<0.05) determined using a two-tailed chi-squared test with one degree of freedom (-) indicates that no statistical analysis was performed between the two cycles, as the difference was considered minor and insignificant for the study's primary outcomes

	Cycle 2	Cycle 3	χ2	p-value
Number of Patients (n)	59	50	-	-
Tourniquet Mention, n (%)	50 (85)	50 (100)	8.314	0.0039*
Skin Assessment Pre, n (%)	0 (0)	28 (56)	44.461	0.0001*
Skin Assessment Post, n (%)	0 (0)	30 (60)	48.843	<0.0001*
Isolation Method, n (%)	0 (0)	30 (60)	48.843	<0.0001*
Exsanguination Method, n (%)	1 (2)	12( 24)	12.819	0.0003*
Tourniquet Pressure, n (%)	23 (39)	38 (76)	15.048	0.0001*
Tourniquet Time, n (%)	35 (59)	47 (94)	17.465	0.0001*

In both audit cycles, the difference between systolic blood pressure (SBP) and documented tourniquet pressure was calculated where documentation was explicit. This excluded a significant subgroup (n=36, 61%) of cycle 2 due to inadequate documentation of tourniquet pressures.

The use of BOAST recommended maximum pressures increased between the two cycles from 17.4% to 63.1% (p=0.0005). Lower limb tourniquet pressures were significantly reduced between the two audit cycles (independent samples t-test, mean: 287.06, SD: 21.73 vs mean: 265.91, SD: 23.84, p = 0.007). However, upper limb tourniquet pressures remained relatively consistent between both cycles (independent samples t-test, mean: 236.67, SD: 21.60 vs mean: 236.56, SD: 26.89, p = 0.993), as outlined in Table 5.

**Table 5:** Overall BOAST compliance with recommended pressures BOAST: British Orthopaedic Association Standards for Trauma (*) significant p-value (<0.05) using independent samples t-test (**) significant p-value (<0.05) determined using a two-tailed chi-squared test with one degree of freedom

	Cycle 2	Cycle 3	p-value
No. of Patients with Documented Pressures (Upper Limb: Lower Limb)	23 (6:17)	38 (16:22)	0.323
Upper Limb Tourniquet Pressures (mean, SD)	236.67 ± 21.60	236.56 ± 26.89	0.993
Lower Limb Tourniquet Pressures (mean, SD)	287.06 ± 21.73	265.91 ± 23.84	0.007*
Tourniquet Pressure Used Within Standard, n (%)	4 (17.4)	24 (63.1)	0.0005**

No tourniquet-related complications were reported in any case in any audit cycle.

## Discussion

The lack of awareness regarding the BOAST guidelines can be attributed to several factors. Tourniquet practices are often based on anecdotal or traditional methods rather than evidence-based medicine or formal standards. Surgeons typically learn about pneumatic tourniquet use informally from senior colleagues rather than through structured education. Although tourniquet use is included in the trauma and orthopaedic intercollegiate surgical curriculum, formal teaching on the topic is notably absent [7]. Additionally, instruction manuals, when available, are rarely reviewed. Our study found that only 44% (n=22) of operating surgeons were aware of the BOAST guidelines, and only 54.8% (137 out of 250) were aware of specific elements that need to be documented. This highlights significant gaps in formal training and reliance on informal instruction. Similarly, in Sadri et al.'s study, a questionnaire given to 54 theatre staff revealed that orthopaedic registrars had a mean understanding score of 41.3% [8], aligning with our findings.

Our audit identified significant gaps in operating surgeons' awareness of documenting skin site conditions (n=14, 28%) and isolation methods (n=14, 28%). Before our intervention, operative records completely omitted these details, mirroring findings from similar studies where 0% of records documented them [9]. One plausible reason is the assumption that nursing staff handle such documentation, making surgeons think it's unnecessary to include it in operative notes. Additionally, the perceived uniformity of procedures within the department may lead to the oversight of these elements, assumed to be common knowledge. However, documentation rates improved notably after we implemented our smart phrase. Skin assessment documentation pre- and post-tourniquet application increased from 0% (n=0) to 56% (n=28) and from 0% (n=0) to 60% (n=30), respectively (p<0.0001), and isolation methods documentation rose from 0% (n=0) to 60% (n=30) (p<0.0001). Another closed-loop audit showed similar improvement, with tourniquet skin status documentation increasing to 69% post-intervention [10], closely aligning with our findings.

In our survey, a high percentage of surgeons were aware of the necessity to document tourniquet pressure (80%, n=40) and duration (94%, n=47). Pre-intervention, tourniquet time was noted in 59% (n=35) of operation notes, similar to a comparable study at 58% [11]. Post-intervention, this documentation increased to 94% (n=47). Documentation of tourniquet pressures improved significantly from 39% (n=23) to 76% (n=38), although this fell short compared to other institutions where pressures were recorded in 89.8% to 94% of cases post-intervention [10,12]. Compliance with BOAST-recommended pressures increased substantially from 17.4% (n=4) to 63.1% (n=24), closely paralleling findings from a similar study in a West Midlands NHS Trust, which showed an increase from 25% to 75% [10]. These results surpassed those of other hospitals, where only 23% of cases met the recommended pressure limits post-intervention [12]. The variation in compliance may be due to the lack of specificity in BOAST guidelines regarding the timing of systolic blood pressure documentation. Initially, tourniquet pressures were often set based on surgeon preference rather than BOAST recommendations, a practice inherited through mentorship. Younger et al. observed that foot and ankle surgeons often preferred tourniquet pressures outside recommended ranges, with only 7% considering systolic blood pressure when selecting tourniquet pressure [13]. Our intervention involved department-wide education and the implementation of a smart phrase to prompt surgeons to calculate tourniquet pressures per BOAST guidance before each case. This led to a significant decrease in mean tourniquet pressure for lower limbs (from 287.06 mmHg to 265.91 mmHg, p=0.007), while upper limb pressures remained similar (236.67 mmHg vs. 236.56 mmHg, p=0.993). These tourniquet pressures align with the Hammond et al. study, which reported mean pressures around 262 mmHg for lower limbs and 237 mmHg for upper limbs [14], highlighting the efficacy of standardised pressure calculation protocols in enhancing adherence to guidelines. It is worth noting that new-generation tourniquets, which can adapt pressure according to the patient's current systolic pressure, are increasingly available and may offer additional benefits. Furthermore, sterile, disposable tourniquet systems that are applied through limb exsanguination can comply with BOAST guidelines, provided they are approved by regulatory bodies.

The introduction of an EPR in September 2022 led to a significant drop in BOAST guideline compliance, from 38% (n=19) to 17.4% (n=10). Challenges such as time constraints and adapting to the new system likely impacted the quality of operative notes, underscoring the need for comprehensive training and familiarisation with new technologies before implementation. Training completion post-rollout may have resulted in undertraining, particularly among older professionals. Additionally, the timing of guideline publication during the COVID-19 crisis may have diverted attention, further impeding adaptation. Despite initial challenges, compliance with BOAST guidelines increased significantly from 17.4% (n=10) to 62.0% (n=31) following enhanced staff proficiency with the EPR system and the introduction of an auto-text proforma with drop-down options (p=0.0005). These findings highlight the effectiveness of proactive training and continued support in overcoming technology transition obstacles, emphasising the importance of perseverance and adaptation in integrating new systems into healthcare practices.

EPRs are vital in modern orthopaedics, offering significant benefits despite adding complexity to the surgeon's workflow. Our study highlights how EPRs can enhance the quality of clinical documentation. A randomised controlled study of 303 notes found that electronic admission documentation showed that the use of electronic notes encouraged the clinician to provide more detailed information in the free-text content compared to paper notes [15]. Orthopaedic-tailored EPRs can meet the unique documentation needs of surgical and clinical practices in line with national standards like BOAST. However, they may contribute to provider burnout, especially among older physicians transitioning to digital tools [16]. Younger physicians, comfortable with technology, experience less burnout and adapt more easily. This suggests that their growing proficiency will drive further technological advancements and efficiencies in clinical settings [16].

Study limitations include the small sample size of 159 patients, which may limit the robustness of the findings, particularly regarding different tourniquet practices. Additionally, poorly recorded results during the first audit loop restricted data interpretation for subsequent cycles. Rotational staff and inconsistent participation in departmental meetings may have hindered the dissemination of audit findings and best practices among operating surgeons, potentially affecting study outcomes.

## Conclusions

This closed-loop audit has demonstrated that implementing a new electronic patient record system initially presents challenges in maintaining accurate operative records. However, through the integration of specialised electronic templates, personalised blood pressure calculations and active staff engagement, significant improvements in tourniquet safety and adherence to best practices can be achieved. As the NHS progresses towards a paperless system, orthopaedic units must take an active role in designing and implementing these systems in their hospitals. By doing so, they can elevate care standards and mitigate potential complications effectively.
